# Implementation and Evaluation of a Grip Behavior Model to Express Emotions for an Android Robot

**DOI:** 10.3389/frobt.2021.755150

**Published:** 2021-10-13

**Authors:** Masahiro Shiomi, Xiqian Zheng, Takashi Minato, Hiroshi Ishiguro

**Affiliations:** ^1^ Advanced Telecommunications Research Institute International, Kyoto, Japan; ^2^ Graduate School of Engineering Science, Osaka University, Osaka, Japan; ^3^ Guardian Robot Project, RIKEN, Kyoto, Japan

**Keywords:** social touch, human-robot interaction, emotional touch, affective touch, haptics

## Abstract

In this study, we implemented a model with which a robot expressed such complex emotions as heartwarming (e.g., happy and sad) or horror (fear and surprise) by its touches and experimentally investigated the effectiveness of the modeled touch behaviors. Robots that can express emotions through touching behaviors increase their interaction capabilities with humans. Although past studies achieved ways to express emotions through a robot’s touch, such studies focused on expressing such basic emotions as happiness and sadness and downplayed these complex emotions. Such studies only proposed a model that expresses these emotions by touch behaviors without evaluations. Therefore, we conducted the experiment to evaluate the model with participants. In the experiment, they evaluated the perceived emotions and empathies from a robot’s touch while they watched a video stimulus with the robot. Our results showed that the touch timing before the climax received higher evaluations than touch timing after for both the scary and heartwarming videos.

## 1 Introduction

For social robots that interact with people, emotional expression is becoming necessary to be accepted by people. For this purpose, robotics researchers developed various kinds of robots that can express emotions using facial expressions (Hashimoto et al., 2006; Glas et al., 2016; Cameron et al., 2018; Ghazali et al., 2018), full-body gestures (Venture and Kulić, 2019; Yagi et al., 2020), and voice (Lim et al., 2012; Crumpton and Bethel, 2016). Affective touch is also an essential part of expressing emotions for human beings (Lee and Guerrero, 2001; Hertenstein et al., 2009; Field, 2010). Due to the advancement of touch interaction-related research works, robots have also acquired the ability to express emotions through touch interactions. For example, a past study investigated how participants touch a robot when they express emotions to elucidate the relationships between the touched parts of the robot and the emotions expressed by people (Alenljung et al., 2018). Other past studies investigated the relationships between facial expressions and touch characteristics to express a robot’s emotions and intimacy with their interacting partners (Zheng et al., 2019a; Zheng et al., 2019b). Another study investigated the effects of the warmness of a robot’s touch toward creating social warmth (Willemse et al., 2018), and the effects of robot-initiated touch behaviors were also investigated in the context of affective and behavioral responses (Willemse et al., 2017). These studies showed the importance and usefulness of affective touch interactions for social robots to convey their emotions.

However, the above studies mainly focused on expressing relatively simple emotions, such as happiness and sadness, which are defined as basic emotions (Ekman and Friesen, 1971). Touch characteristics, such as type and place, are essential to express such basic emotions (Zheng et al., 2019a; Zheng et al., 2019b). Although for expressing complex emotions (i.e., heartwarming and horror feelings that combine multiple simple emotions), we need to consider such time-dependent features as touch timing and longer durations. For example, past studies reported that a heartwarming emotion lasts relatively longer than a negative emotion after the former is evoked (Tokaji, 2003; Takada and Yuwaka, 2020). Another past study focused on modeling appropriate touch timing and duration to express such complex emotions, but they did not evaluate our models (Zheng et al., 2020). In other words, there is room to investigate the effectiveness of the affective touches of social robots with complex emotions.

Based on our previous study (Zheng et al., 2020), this new study implements and evaluates a touch behavior model that expresses heartwarming and horror emotions with a robot, i.e., this paper is a follow-up study of Zheng et al., 2020. We used an android named ERICA (Glas et al., 2016) to implement our developed model. We also conducted an experiment with human participants to evaluate its effectiveness in the context of expressing both heartwarming and horror emotions by the robot.

## 2 Materials and Methods

**FIGURE 1:**
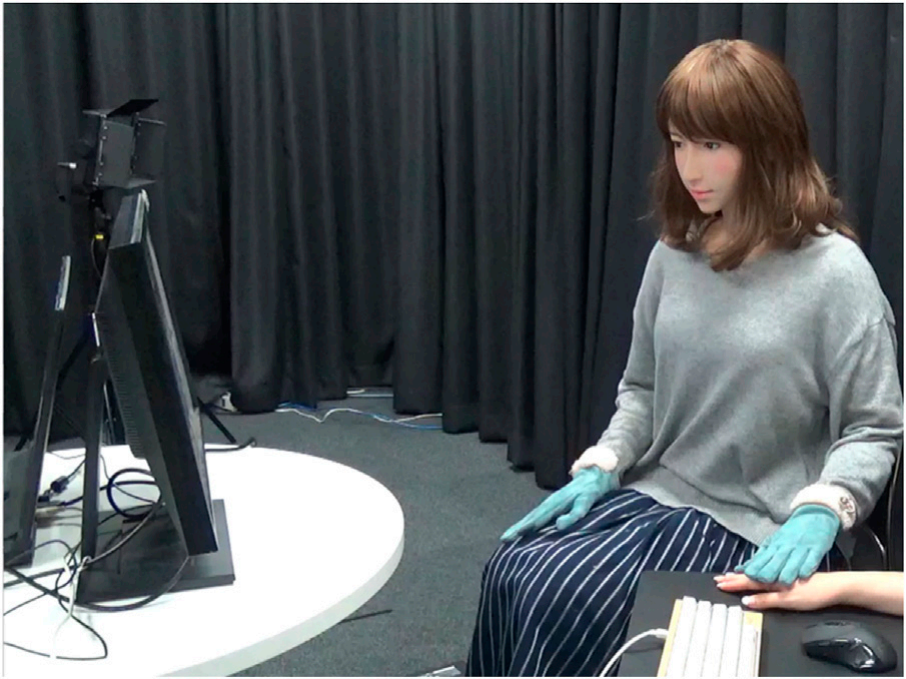
Participant and ERICA watch a video.

This study used similar materials and methods from our past study (Zheng et al., 2020) that modeled touch behaviors to express heartwarming and horror emotions. The participants identified appropriate touch (grip) timing and durations using a robot in the data collection of our previous work. Thus, the participants adjusted the timing and duration of the robot’s touch behavior by themselves to reproduce a natural feeling while watching heartwarming and horror video stimuli together (Figure 1). We note that such video-induction settings are effective in arousing specific emotions (Lithari et al., 2010; Wang et al., 2014). In other words, the participants designed touch behaviors that provide the contextually relevant emotional feeling as a natural touch behavior. We used a fitting approach for probabilistic distribution based on the gathered data and modeled the touch behaviors to express heartwarming and horror emotions.

### 2.1 Target Emotions and Video Stimulus

We focused on heartwarming and horror emotions as target emotions that our past study also targeted (see II.A of Zheng et al., 2020). We focused on positive emotion because past related studies also focused on positive emotion expressions to build a positive relationship with interacting people (; Ekman, 1993; Fong et al., 2003; Kanda et al., 2007; Weining and Qianhua, 2008; Bickmore et al., 2010; Kanda et al., 2010; Leite et al., 2013a; Leite et al., 2013b; Tielman et al., 2014; Wang and Ji, 2015; Rossi et al., 2017; Cameron et al., 2018) . To investigate whether our implementation approach is useful to reproduce a different kind of emotion, we focused on a negative emotion as a counterpart to the target (positive) emotion. These emotions consist of multiple basic emotions; past studies with Japanese participants reported that Japanese people feel happy and sad under deeply heartwarming situations (Tokaji, 2003). Horror is an intense feeling that combines fear and surprise or disgust. Note that our study experimented with Japanese participants and evaluated these two emotions as target feelings with them.

We also used video stimuli to provoke these emotions based on our past study (Zheng et al., 2020), which used six video clips (three each for heartwarming and horror) from YouTube^1^ to gather data about touch characteristics. Although the settings of our experiment are different from Zheng et al. (2020), we believe that using all identical video stimuli would be unsatisfactory for investigating the generality of our implemented models. Therefore, we mixed a part of the original videos with new video stimuli for our evaluation in this study and used eight commercially available videos from YouTube. Four videos (two heartwarming/horror), which are identical materials from the original study, and four new videos (two heartwarming and two horror videos) for this study.^2^ Thus, we used eight videos in total.

### 2.2 Robot Hardware

We used an android with a feminine appearance, ERICA (Glas et al., 2016), which was also used in our past study. All of her hardware configurations are identical as in the past study (Section II.B of Zheng et al., 2020). The frequency of her motor control system for all the actuators was 50 ms. ERICA wore gloves to avoid mismatched feelings between appearance and touch. Her skin is a silicone-based design even though its appearance is human-like, whose touch feeling is quite different from human’s hand. The difference between the actual feeling and the feeling evoked by the appearance may cause strong discomfort. In order to reduce this effect, we put on gloves to avoid any discomfort.

### 2.3 Implementation of Touch Behavior

Our experiment follows the same setting to design the touch behaviors as in the past study (II.C of Zheng et al., 2020). The robot grips the participant’s right hand with its left hand by closing her five fingers while they are watching video stimuli together. We re-implemented our proposed model because the original paper simply implemented it in the robot and only checked its timings and durations.

**FIGURE 2:**
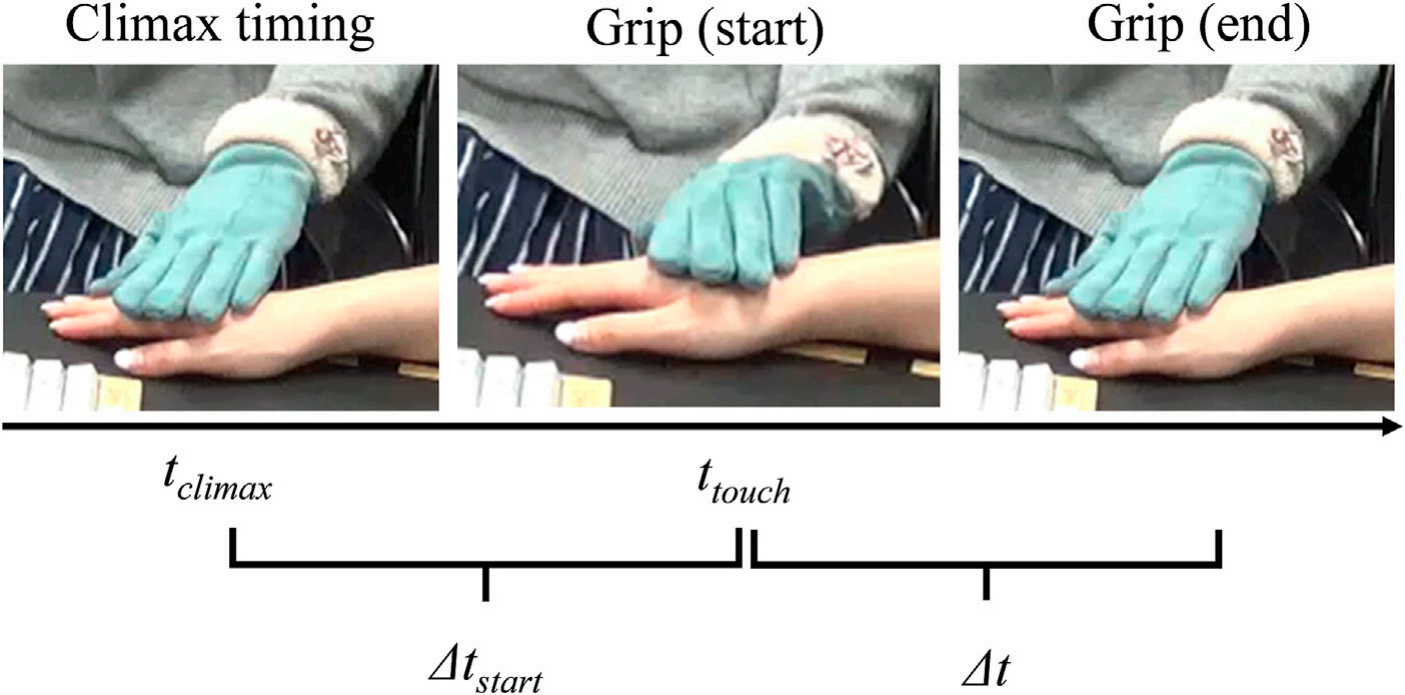
Illustration of t_climax_, t_touch_, ∆t, and ∆t_start_.

The implementation process requires four kinds of parameters: 1) the most appropriate climax timing of that video, t_climax_; 2) the timing at which the robot should start its grip as a reaction (or anticipation) to the climax, t_touch_; 3) the grip’s duration ∆t; and 4) ∆t_start_ (i.e., t_climax_—t_touch_), which is the difference between the touch and climax to extract the timing features (Figure 2). The reason for using grip behaviors is that a past study succeeded in conveying emotions *via* a grip behavior (Cabibihan and Chauhan, 2017).

**FIGURE 3:**
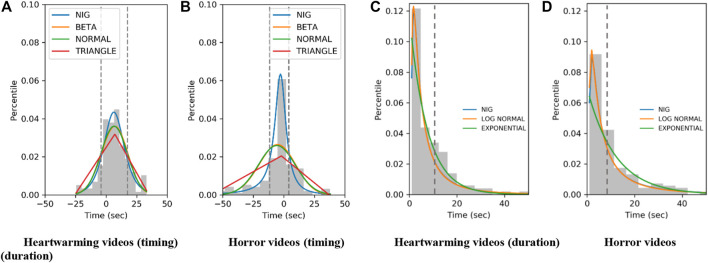
Histograms of grip timing and duration and fitting results with functions. **(A)** Heartwarming videos (timing). **(B)** Horror videos (timing). **(C)** Heartwarming videos (duration). **(D)** Horror videos (duration).

Our past study concluded that using a normal-inversed Gaussian (NIG) function is a better approach to model t_start_ and ∆t compared to other functions (e.g., beta, normal, triangle, log-normal, and exponential). Therefore, we used the NIG function in this study, and it showed the best fitting results for implementation. Figure 3 shows the histograms and the fitting results with the NIG functions, which used the defined parameters by Zheng et al. (2020) [the detailed information about the parameters are written in Zheng et al. (2020)].

One concern from the implementation perspective is the relatively large standard deviations of the models, which we did not previously discuss (Zheng et al., 2020). In extreme cases, 24.53–32.99 and 55.37–37.85 are the possible ∆t_start_ ranges of the heartwarming and horror NIG models. Directly sampling from these ranges might fail to reproduce typical touch timings based on gathered data, i.e., reacting before the horror climax or after the heartwarming climax. We mitigated this problem by limiting the sampling range within one standard deviation (dotted lines in Figure 3). If a sampled value falls outside the range, we sample it again until the value is within one standard deviation from the mean. Although this approach is rather ad-hoc, it effectively reproduced typical touch parameters based on gathered data. Based on this implementation policy, the possible ∆t_start_ ranges of the heartwarming and horror NIG models are −4.37–17.42 and −11.55–4.13 s. We note that t_climax_ is pre-defined for each video based on the data collection results; it is used for calculating the t_touch .=_ t_climax +_ ∆t_start._

For selecting touch duration ∆t, we re-analyzed the touch data within one standard deviation sampling range due to the above implication policies. First, 11 of 11 touches in the heartwarming videos and 49 of 59 touches in the horror videos started before the climax timing and ended after it, i.e., a similar touch characteristic from the original data. We again conducted a binominal test and found significant differences between them (heartwarming: *p* < 0.001, horror: *p* < 0.001). However, using a sampling method might fail to reproduce typical touch parameters. The majority of touches started before the climax timing and ended after it. But due to the likelihood of exceedingly small touch durations, for example, a sampled minus −3 s ∆t_start_ and a sampled 1-s ∆t mean the touch will end 2 s before the climax. In fact, even though we limited the sampling ranges, ∆t’s sampling ranges are still relatively wide to reliably reproduce the typical touch parameters. To reproduce them, we simply used the mean values instead of the sampling approach, i.e., ∆t is 8.525 s for the heartwarming videos and 12.857 s for the horror videos. Based on this calculation, the system calculates the end timing of robot’s grip (t_touch +_ ∆t).

## 3 Experiment

### 3.1 Hypotheses and Predictions

Past studies investigated what kinds of touch behaviors effectively conveyed emotions in human-robot touch interaction and provided rich knowledge that contributed to the design of robot behaviors (Lee and Guerrero, 2001; Hertenstein et al., 2009; Field, 2010; Zheng et al., 2019a; Zheng et al., 2019b). Unfortunately, appropriate touch timings and their durations to convey emotions have received insufficient focus.

To identify appropriate touch timings and durations in the context of conveying emotions, we previously implemented touch timing and duration models based on the data collection of human-robot touch-interaction settings (Zheng et al., 2020). According to our proposed model, touching before the climax with a relatively long duration is suitable for expressing horror, and touching after a climax with a relatively short duration is suitable for heartwarming emotion. If the modeling is appropriate, a robot’s touch that follows the model will be perceived as more natural than disregarding the model.

Moreover, at the data collection in the past study (Zheng et al., 2020), the robot’s grip behaviors are designed by considering not only conveying emotions but also showing empathy following the instructions. Another past study reported that touch parameters such as length and frequency have influenced of effects of empathic touch between humans and robots. Therefore, if the modeling is appropriate, the participants will feel that they and the robot empathize with each other (Bickmore et al., 2010). Based on these hypotheses, we made the following three predictions:

Prediction 1 If the robot touches the participant using the heartwarming NIG model when it is watching heartwarming videos with the participants, its touch will be perceived as more natural than a robot that uses the horror NIG model.

Prediction 2 If the robot touches using the horror NIG model when it is watching horror videos with the participants, its touch will be perceived as more natural than a robot that uses the heartwarming NIG model.

Prediction 3 If the robot touches with a NIG model for videos in the same category, the participants will feel that they and the robot empathized with each other.

### 3.2 Participants

We recruited 16 people (eight females and eight males) whose ages ranged from 21 to 48 and averaged 34. They had diverse backgrounds, and none joined our previous data collection (Zheng et al., 2020).

### 3.3 Conditions

Our experiment had a within-participant design. Each participant experienced the four conditions under two factors described below (*category* factor: *heartwarming video* and *horror video* and *model* factor: *heartwarming* NIG and *horror* NIG). The category factor is related to video stimuli, and the model factor is related to the robot’s behaviors. For example, in the combination of *heartwarming video* (the category factor) and *heartwarming* NIG (the model factor), the participant will watch heartwarming videos, and the robot will grip the participant based on the heartwarming NIG function.

#### 3.3.1 Category Factor

This factor has two video conditions: *heartwarming* and *horror*. In the *heartwarming* video condition, the participants and the robot watched heartwarming videos together. In the *horror* video condition, they watched horror videos together. As described in Section 2.1, we downloaded eight videos from YouTube. Four videos (two heartwarming/horror) are identical materials from our previous data collection. We selected four new videos (two heartwarming/horror videos) for this experiment.

#### 3.3.2 Model Factor

This factor also has two NIG conditions: *heartwarming* and *horror*. In the *heartwarming* NIG condition, the robot samples from one standard deviation range of the *heartwarming* NIG, and in the *horror* NIG condition, it samples from the horror NIG, described in Section 2, to determine the touch-timing characteristics.

Since the robot needs to know the t_climax_ for each video, we conducted a preliminary survey for these eight videos to control the touch timing in both conditions. Fifteen participants from our institutions (without any knowledge of our study), whose ages ranged from 24 to 35 and averaged 26, provided their perceived climax timing of each video. We used the average of the largest clusters of the histograms of the climax timing as t_climax_ for each video. We edited the videos to only have one typical climax timing (i.e., extracted t_climax_ for the robot) and at least another 30 s after the climax to the end of the video to leave enough time to finish the robot’s touch.

### 3.4 Measurements

To compare and investigate the perceived naturalness of ERICA’s touch behaviors, the participants compared two aspects in the first questionnaire: Q1) naturalness of touch (degree of naturalness of touch behavior to express emotion) and Q2) naturalness of touch timing (degree of naturalness of touch timing). Participants answered this questionnaire for each video.

We also asked the participants about their perceived empathy with ERICA from two aspects in the second questionnaire: Q3) perceived empathy to ERICA (degree of perceived empathy to ERICA) and Q4) perceived empathy from ERICA (degree of perceived empathy of ERICA to you). Participants answered this questionnaire after each condition (i.e., once for each condition). We used a response format on a seven-point scale for these questionnaires, i.e., describing the options ranging from most negative to most positive.

In the second questionnaire as a manipulation check, we asked the participants about their perceived emotions from the robot’s touch. The only emotional signal from the robot is her grip behavior; she said nothing throughout the entire experiment and maintained a neutral facial expression. Although we expected the perceived emotions to reflect the category factor, confirming them is important. We asked the participants to select the top two perceived emotions (Q5/Q6) from Ekman’s basic six emotions (Ekman, 1993). The participants selected one emotion from six candidates by using radio buttons.

### 3.5 Procedure

Before the experiment, the participants were given a brief description of its purpose and procedure. Our institution’s ethics committee approved this research for studies involving human participants (20-501-4). Written, informed consent was obtained from them.

First, we explained that they would be watching a series of heartwarming/horror videos with ERICA, who would continue to touch their hand during the process. Sometimes she would grip it to convey emotion. The participants sat on ERICA’s left. To reproduce identical touch behaviors for all the participants, they put their right hands on specific table markers to guarantee that they all experienced identical interactions during the identical touch behaviors, and we asked the participants to use the mouse with their left hand to start the video stimuli. To avoid discomfort feeling due to constraining their hand position, we adjusted the height of the chair.

After the experiment started, the participants used a user interface to play the videos. Each video played independently. Before starting each video, the robot calculates t_touch_ and ∆t based on the t_climax_ of each video by using the NIG function. The used parameters are different due to the condition; the robot uses the parameters for the heartwarming model under the *heartwarming* NIG condition or the horror model under the *horror* NIG condition. Because we used a probabilistic distribution approach, the grip timing and duration are different between the participants even though they watched the same video. When a video started, ERICA put her hand on a participant’s hand and gripped at a selected moment that lasted a certain duration using the mechanism described in Section 2. After each video, ERICA resumed her default pose (i.e., ERICA’s hand would leave the participant’s hands), and the UI showed Q1 (“Do you feel ERICA’s touch naturally conveys her emotions?”) and Q2 (“Do you feel ERICA’s touch timing is natural?”) and radio buttons with texts (1: most negative, 7: most positive) to evaluate them. When the video was the final stimulus (i.e., the fourth video) for each condition, the UI showed the second questionnaire from Q3(“Did you feel sympathy to ERICA?”), Q4 (“Did you feel whether ERICA feels sympathy to you?”) and the same radio buttons with the texts to evaluate them. In addition, the UI showed Q5 (“Which emotion is the top perceived emotion from ERICA?”) and Q6 (“Which emotion is the second top perceived emotion from ERICA?”) and radio buttons with texts about the name of basic emotions.

We adopted a counterbalanced design for each factor. The order of the category factor is randomized, and all the horror or heartwarming videos were played randomly, during which ERICA drew samples from either the horror or the heartwarming NIG. Then the videos were played again as ERICA drew samples from the second NIG. These steps were repeated for the remaining videos.

## 4 Results

### 4.1 Manipulation Check

Table 1 shows the integrated number of perceived emotions from Q5/Q6. The total number for each NIG is 32 because we asked about two perceived emotions, which depended (by touching) on the category factor. The majority of the perceived emotions for the *heartwarming* and *horror* categories are happy/sad and fear/surprise, regardless of the NIG functions. For the former category, similar to a past study that investigated expressions of deeply heartwarming emotion in Japan (Tokaji, 2003), the participants selected happy and sad emotions as perceived emotions. For the latter category, participants reported typical emotions, i.e., surprise and fear about horror videos. The results showed that most participants felt happy/sad or fear/surprise toward heartwarming and horror videos as we expected.

**TABLE 1:** Perceived emotion from robot’s touch.

Heartwarming videos	Happy	Sad	Surprise	Fear	Disgust	Anger
Heartwarming NIG	16	15	1	0	0	0
Horror NIG	16	15	1	0	0	0
**Horror videos**	**Happy**	**Sad**	**Surprise**	**Fear**	**Disgust**	**Anger**
Heartwarming NIG	0	0	10	16	6	0
Horror NIG	0	0	15	16	1	0

### 4.2 Verification of Predictions 1 and 2

**FIGURE 4:**
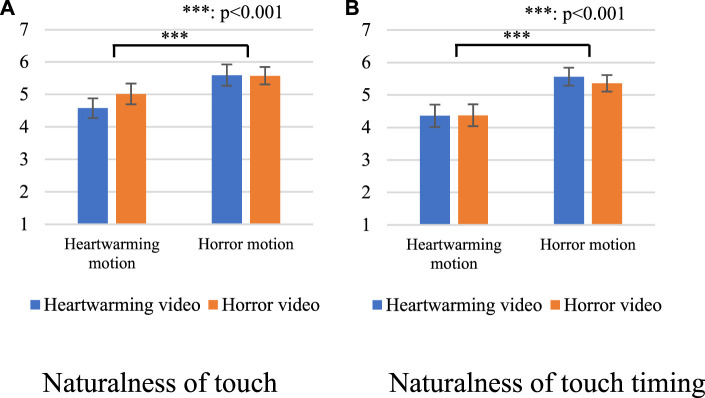
Questionnaire results with all videos. **(A)** Naturalness of touch. **(B)** Naturalness of touch timing.

Figure 4A shows the questionnaire results of the naturalness of touch. We conducted a two-way analysis of variance (ANOVA) for each factor on *category* and *model*. The sphericity of the analysis was not violated in this setting. We identified a significant main effect in the *model* factor [F(1,15) = 16.736, *p* < 0.001, partial *η* ^2^ = 0.527]. We did not identify a significant main effect in the *category* factor [F(1,15) = 1.306, *p* = 0.271, partial *η* ^2^ = 0.080] or in the interaction effect [F(1,15) = 1.823, *p* = 0.197, partial *η* ^2^ = 0.108].

Figure 4B shows the results of the naturalness of the touch timing. We conducted a two-way ANOVA for each factor on *category* and *model*. The sphericity of the analysis was not violated in this setting. We identified a significant main effect in the *model* factor (F(1,15) = 47.481, *p* < 0.001, partial *η* ^2^ = 0.760). We did not identify a significant main effect in the *category* factor [F(1,15) = 0.148, *p* = 0.706, partial *η* ^2^ = 0.010] or in the interaction effect [F(1,15) = 575, *p* = 0.021, partial *η* ^2^ = 0.328].

These results show that the participants evaluated the touches higher with the horror NIG regardless of the video categories. Thus, **Prediction 2** was supported, but not **Prediction 1**.

### 4.3 Verification of Prediction 3

**FIGURE 5:**
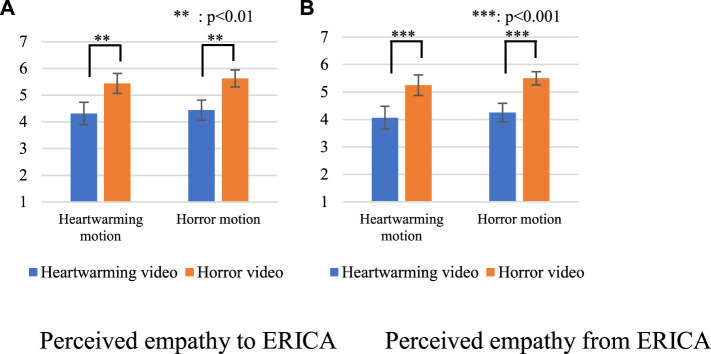
Questionnaire results about perceived empathy. **(A)** Perceived empathy to ERICA. **(B)** Perceived empathy from ERICA.

Figure 5A shows the results of the perceived empathy to ERICA. We conducted a two-way ANOVA for each factor on *category* and *model*. The sphericity of the analysis was not violated in this setting. We identified a significant main effect in the *category* factor [F(1,15) = 9.765, *p* = 0.007, partial *η* ^2^ = 0.394]. We did not identify a significant main effect in the *model* factor [F(1,15) = 0.256, *p* = 0.620, partial *η* ^2^ = 0.017] or in the interaction effect [F(1,15) = 0.016, *p* = 0.900, partial *η* ^2^ = 0.001].

Figure 5B shows the results of the perceived empathy from ERICA. We conducted a two-way ANOVA for each factor on *category* and *model*. The sphericity of the analysis was not violated in this setting. We identified a significant main effect in the *category* factor [F(1,15) = 21.626, *p* < 0.001, partial *η* ^2^ = 0.590[. We did not identify a significant main effect in the *model* factor [F(1,15) = 0.852, *p* = 0.371, partial *η* ^2^ = 0.054] or in the interaction effect [F(1,15) = 0.028, *p* = 0.868, partial *η* ^2^ = 0.002].

These results show that participants felt empathy with the robot when they watched horror videos, regardless of the NIG models. Thus, **Prediction 3** was not supported.

## 5 Discussion

### 5.1 Additional Analysis of Model’s Validity

We applied two video stimuli in each video category and used them in the data collection experiment in our verification experiment. We did not think combining the old and new video stimuli were problematic because the participants who trained the models in the first experiment and evaluated them in the second experiment differed. Evaluating the models with only new video stimuli would provide additional evidence of effectiveness.

**FIGURE 6:**
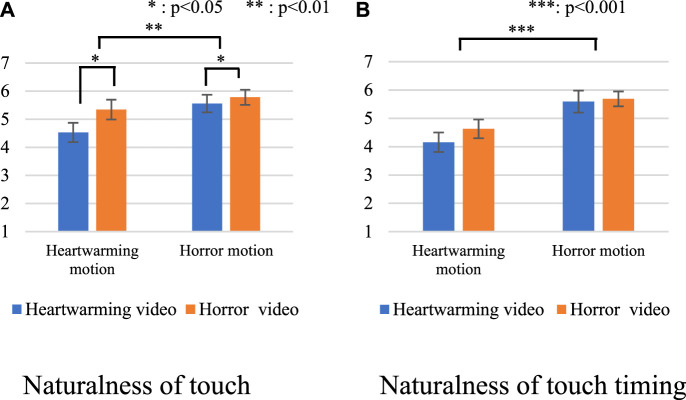
Questionnaire results with only new videos. **(A)** Naturalness of touch. **(B)** Naturalness of touch timing.

Figure 6A shows the questionnaire results of the naturalness of touch with only new videos. We conducted a two-way ANOVA for each factor on *category* and *model.* The sphericity of the analysis was not violated in this setting. We identified significant main effects in the *model* factor [F(1,15) = 13.720, *p* = 0.002, partial *η* ^2^ = 0.478] and in the *category* factor [F(1,15) = 6.505, *p* = 0.022, partial *η* ^2^ = 0.303]. We did not identify a significant main effect in the interaction effect [F(1,15) = 2.517, *p* = 0.133, partial *η* ^2^ = 0.144].

Figure 6B shows the questionnaire results of the naturalness of the touch timing for only new videos. We conducted a two-way ANOVA for each factor on *category* and *model.* The sphericity of the analysis was not violated in this setting. We identified a significant main effect in the *model* factor [F(1,15) = 30.612, *p* < 0.001, partial *η* ^2^ = 0.671]. We did not identify a significant main effect in the *category* factor [F(1,15) = 1.086, *p* = 0.314, partial *η* ^2^ = 0.068] or in the interaction effect [F(1,15) = 0.789, *p* = 0.388, partial *η* ^2^ = 0.050]. These results show the models are effective for the video stimuli that were not used in the data collection. We note that the statistical analysis for only the videos in the data collection showed similar trends.

### 5.2 Design Implications

Unlike our hypotheses, the experiment results showed a better impression invoked by the horror NIG model where the robot and the participants watched both heartwarming and horror videos together. This result provides design implications for a robot’s touch behavior.

First, as a touch behavior implementation for a social robot, touch timing before a climax provides better impressions than touch timing after it, at least in a touch-interaction scenario where videos were the only external emotional stimuli that people intended to watch with the robot. One technical consideration is how to estimate the climax timing. Several past studies proposed methods to identify highlighted movie scenes by information processing (Weining and Qianhua, 2008; Wang and Ji, 2015). Such an approach might be useful to define climax timing for videos.

Second, our result suggests that directly using the parameters observed from human behaviors might overlook better parameters for the behavior designs of robots in emotional interaction contexts. From our study, even with an abundant number of participants for data collection, the observed touch models, i.e., heartwarming NIG, failed to reflect the people’s actual expectations in the evaluations.

Why did the observed heartwarming NIG model show disadvantages? Perhaps the setting was different between the data collection and the experiment. During the data collection, our participants watched the videos repeatedly to identify the robot’s touch-timing characteristics. They already knew the climax timing of the video. On the other hand, they had no prior knowledge about the video stimuli or their climax timing in the experiment. In such situations, perhaps the touch timing before the possible climax was interpreted more favorably because such touch timing might demonstrate a sense of empathy to people who were touched in this way. Another possibility is that the climax timing was different between the system and participants because the variance of heartwarming videos is relatively larger than the horror videos. For example, if the climax timing of the participants were earlier than the system’s timing, perceived naturalness becomes lower.

In addition, even though the estimated emotions by participants matched the video categories, we identified no significant effects for perceived empathy. The expressions of emotions only by touching might be implicit. To perceive empathy, feeling, and sharing another’s emotions are important. Therefore, such implicit expressions might be insufficient to increase the perceived empathy. As we previously discussed (Zheng et al., 2020), using different modalities to express emotions might effectively increase the perceived empathy explicitly.

Another point of view is the category of the contents for the shared experiences. Our results did not show any significant effect of our touch behavior design toward perceived empathy, although the participants perceived higher empathy when they watched the horror video stimuli than the heartwarming video stimuli. Moreover, participants felt happy/sad and fear/surprise for the *heartwarming* and *horror* video stimuli, regardless of the NIG functions, which might indicate that the types of visual stimuli have relatively stronger effects than the touch stimuli toward perceived empathy and emotions. Whether this phenomenon is common regardless of the co-viewer types (e.g., different appearances or beings) remains unknown. Investigating the relationships among perceived empathy, co-viewer’s characteristics, and the categories of co-viewing content are interesting future work.

### 5.3 Limitations

Since we only used a specific android robot with a female appearance, we must test different types of robots before generalizing our experimental results. In addition, our android’s hands resemble human hands and can perform gripping behaviors. For robots without such hand structure, other touch characteristics must be considered. The robot’s appearance and the participant’s ages influence the perceived emotions (Zheng et al., 2020). Moreover, the experiences of the participants, whether they are used to be touched, and the degree of their perceived emotions via visual stimulus would have influences.

We only used heartwarming and horror videos as emotional stimuli because they are typically used in human-robot interaction studies and human science literature. Investigating appropriate touch timing for different emotions is needed to convey such emotions by touching.

## 6 Conclusion

Affective touch is an essential part of expressing emotions for social robots. However, past studies investigated the effectiveness of using touch behaviors to express robot emotions and generally focused on expressing relatively simple emotions. Although our past study (Zheng et al., 2020) focused on modeling appropriate touch timing and duration to express such complex emotions as heartwarming (mixing happiness and sadness) and horror emotions (mixing fear, surprise, and disgust), we did not evaluate our models. Therefore, in this study, we implemented and evaluated our previous touch behavior model in experiments with human participants, which expresses heartwarming and horror emotions with a robot. We modified the normal-inversed Gaussian (NIG) distribution functions proposed in our past study and used an android with a feminine appearance for implementation.

To evaluate the developed models, we experimented with 16 participants to investigate the effectiveness of both models using heartwarming/horror videos as emotional stimuli. The horror NIG model has advantages compared to the heartwarming NIG model, regardless of the video types, i.e., people preferred a touch timing before climax for both videos. This knowledge will contribute to the emotional touch-interaction design of social robots.

## Data Availability

The raw data supporting the conclusion of this article will be made available by the authors, without undue reservation.

## Footnotes

1
https://youtu.be/PFhQhpR5Z8M, https://youtu.be/r1gz-m5Ai_E, https://youtu.be/b2MH-yxIR4Y, https://youtu.be/4LYK0rTjlM8, https://youtu.be/dCPiAOiKSyo, https://youtu.be/gXfLl3qYy0k

2
https://youtu.be/ftaXJlvn5f4, https://youtu.be/cPHLllSvKr8, https://youtu.be/hJxvt5LNnKg, https://youtu.be/7sVl_Mi9d0Q, https://youtu.be/b2MH-yxIR4Y, https://youtu.be/4LYK0rTjlM8, https://youtu.be/dCPiAOiKSyo, https://youtu.be/gXfLl3qYy0k
